# Outcome of inadvertent high dose BCG administration in newborns at a tertiary care hospital, Karachi- Case series

**DOI:** 10.1371/journal.pone.0219324

**Published:** 2019-07-10

**Authors:** Sonia Qureshi, Khalil Ahmad, Paras Fatima, Rabia M. Hassan, Farheen Sherali, Naureen Lalani, Fyezah Jehan, Syed Asad Ali, Farah Naz Qamar

**Affiliations:** 1 Department of Pediatrics and Child Health, Aga Khan University, Karachi, Pakistan; 2 Medical Student, Aga Khan University, Karachi, Pakistan; MCRI, AUSTRALIA

## Abstract

Bacillus Calmette-Guérin (BCG) vaccine is given to newborns soon after birth. BCG vaccine overdose has been rarely reported. Here we report the outcome of newborns who accidently received high dose BCG at a tertiary care hospital, Karachi. We reviewed records of 26 newborns, who accidentally received intradermal high dose BCG, used for the treatment of urinary bladder cancers and 80 times higher dose than the BCG used for routine vaccination. The incident happened from 14-16^th^ April, 2016 at Aga Khan University Hospital, Karachi. Analysis was carried out using SPSS. A total of 23/26(88.5%) newborns were followed for atleast 3 months and 11/26 (42.3%) were followed for atleast one year. 13/26 (50%) were male. All 26 patients were prescribed isoniazid and rifampicin for 3 months. 3/26 (11.5%) were lost to follow-up before completion of anti-tuberculous drugs (ATT). Lesions at the BCG site were observed in 16/26 (61.5%) infants, of which 15 (93.8%) had a papule, 3 (18.8%) developed a pustule, 3 (18.8%) had skin induration and 2 (12.5%) had skin erythema. Axillary lymphadenopathy was observed in 1/26 (3.8%) patient. Coagulation was deranged in 3/26 (11.5%) of babies. Intracranial bleeding was observed in 1/26 (3.8%) case. Localized skin lesions were the most common adverse events. None of them developed clinical tuberculosis. Chemoprophylaxis for inadvertent high dose BCG administration should be given for atleast 3 months. Furthermore, vigilant follow-up, transparency and disclosure are the vital steps in the management of any medical error.

## Introduction

Bacillus Calmette-Guérin (BCG) vaccine, containing live attenuated strain of *Mycobacterium bovis*, is given routinely to newborns as part of the Expanded Program on Immunization in countries with high prevalence of TB. In Pakistan, BCG is given at birth to prevent infection with *Mycobacterium tuberculosis* (TB) and is effective against severe infant tuberculous meningitis and miliary TB [1]. BCG is a safe vaccine but being live attenuated, there is a risk of localized inflammatory reactions (skin erythema, papule, pustule) and axillary lymphadenopathy[2]. Vaccination is followed by development of a papule at injection site within 2–3 weeks of vaccination [3]. There are no universal criterions for defining a BCG adverse reaction. O’Brien et al. have described local swelling, regional lymph node enlargement >20mm, ulcer for greater than 6 weeks as mild complications, ‘axillary abscess or fistula as moderate complications and disseminated BCG infection as severe [4, 5].

Overdose with BCG is rarely reported; with estimated incidence of 2.2 cases per 1 million doses distributed, a likely underrepresentation due to either underutilization of vaccine doses distributed or underreporting of medical errors [6, 7]. There is recent increased use of BCG intravesically at higher doses for immunotherapy management of early-stage bladder cancer [8]. If this is wrongly injected as neonatal BCG, an overdose can occur. It can also occur if an excessive volume is injected or BCG vaccine reconstituted with the inappropriate volumes of diluent [6].

A European report by Lotte et al. in 1984 documented most side effects of BCG overdose following immunization were exaggerated local reactions of large, deep ulcers, necrotic lesions or abscesses, and sometimes regional suppurative lymphadenitis were observed and only 8% developed systemic complications including fever, headache, and malaise [9, 10]. In 1996, 857 infants being injected intradermally with a percutaneous BCG preparation in Manchester which was 5 times the upper limit of the normal dose, 61 (11%) of these infants had adverse local reactions and 48 infants (8.6%) had axillary lymphadenopathy. None of the infant developed disseminated TB [4]. Such accident also reported from Taiwan in 2012 in which the vial was diluted with 2mL instead of 3mL. That vaccine was then administered to 20 healthy infants. 3 (15%) of these infants developed injection site abscesses that resolved within 6 weeks [4]. There have been multiple case reports on BCG overdose in which the authors reported localized reactions only [4, 6, 9].

There is no consensus and large variations in treatment of BCG overdose. Management options have included surgical excision within 12 hours of administration, use of anti-tuberculosis medications, or aspiration of formed abscesses [6]. It is also suggested to follow-up post-therapy to check for any reactivation of local or regional lesions [4]. Here we are reporting the outcome of 26 healthy newborns who were accidentally vaccinated with BCG usually given in patients with bladder cell carcinoma intravesically.

## Material and methods

### Description of the incident

At the Aga Khan University Hospital, 26 newborns born between 11^th^-14^th^ April, 2016 were accidentally vaccinated from 14^th^-16^th^ April, 2016 with 0.05 ml of ONCO-BCG which is used for intravesical instillation in patients with bladder cell carcinoma. The dose given was equivalent to 20 mg, which was 80 times higher than the dose of routine BCG vaccine given at birth. The ONCO-BCG contains a live lyophilized preparation derived from attenuated strain of Mycobacterium bovis. The incident was unconditionally disclosed to the parents as soon as the error was noted. All the newborns were examined and the ones who were already discharged were called back to clinic the next day and examined. These parents were provided with an emergency contact number of a Nurse Manager who could be reached 24/7 for any concerns. The incident was also informed to the pediatricians and gynecologists responsible for the babies. Furthermore, a panel was arranged consisting of pediatric infectious disease specialists, nursing supervisors, pharmacist, departmental chair and hospital ethics committee. Our team also contacted international experts who have dealt with the similar situation on how to best treat and follow up with these patients. There was lack of a prior experience on the management of such events; therefore, a consensus was taken to initiate post-exposure prophylaxis with 10mg/kg/day of isoniazid and 15mg/kg/day of rifampicin for at least 3 months. A stringent protocol of follow-up was planned with an infectious disease specialist. All the babies were followed in Infectious disease clinics at monthly intervals or in between if needed. The cost of the treatment was borne by the hospital for a period of one year.

### Data collection

Records of 26 newborns who had received BCG overdose accidentally from 14^th^ -16^th^ April, 2016 were reviewed retrospectively. Data on local and systemic adverse events such as skin changes including skin erythema, induration, pustules, vesicles, ulcers or abscess formation, osteomyelitis, humeral osteitis, lymphadenitis, pallor, jaundice, intracranial bleeding or disseminated TB, number of follow-up visits and outcome of the newborns was extracted from the medical record files. Statistical analysis was carried out by using SPSS version 17. Frequencies and percentages were reported for categorical variables like gender, ward unit, ATT received, and adverse effects. Means and standard deviations (medians and IQR where appropriate) were reported for age at vaccination, gestational age, duration of ATT, follow-up days and number of clinic visits.

## Results

The median (min- max) age of newborns at vaccination was 2.0 (2.0–4.0) days. 13/26 (50%) were male and 24/26 (92%) were born at full term of gestation. Out of 26 babies 23(88.5%) were followed for at least 3 months and almost half, 11/26 (42.3%) were followed for one year. All 26 patients were prescribed two drugs (isoniazid and rifampicin) anti-tuberculous treatment (ATT) for 3 months. Only 3/26 (11.5%) were lost to follow-up before completion of ATT. However, we have contacted these 3 families on phone; all babies have completed their ATT course and are doing well (Table 1). 3/26(11.5%) babies received ATT for more than 3 months. Localized skin lesions were observed in 16/26 (64%) of newborns. Majority of the localized skin lesions resolved in the first two weeks of life. One newborn developed a persistent weeping skin lesion at the left big toe and underwent skin biopsy at 3 months of age that showed inflammatory changes with eosinophilia. The lesion resolved in 3 months with antiallergy, topical steroids and antibiotics. Coagulation was deranged in 3/26 (11.5%) babies. Out of three babies, 2 presented with post circumcision bleed, intracranial bleeding was observed in 1/26 (3.8%) babies at 37^Th^ day of life when he was admitted with complaints of fever, cough and irritability. During the hospital stay he developed right sided focal seizure. His laboratory investigations revealed hypocalcaemia (serum calcium: 6.5mg/dl), sudden drop in hemoglobin (Hb 6.3 g/dl), had a normal coagulation profile (INR of 1.2), a normal platelet count (427*109/L) and also received vitamin k at birth. Neuroimaging had done which was suggestive of left subdural hematoma and minimal subarachnoid hemorrhage. Considering normal coagulation profile factor XIII deficiency was also ruled out (Factor XIII: 102% (Normal: 70%-140%). He was managed conservatively with calcium supplementation, vitamin K, antiepileptic, antibiotics and packed cell transfusion. He was discharged in stable condition and remained well on antiepileptic with no focal neurologic deficit. After a period of one year, neuroimaging showed resolution of hemorrhage. However, he achieved his developmental milestones late according to his age. None of the patients developed fever, osteomyelitis and clinical or disseminated TB. (Tables 2 and 3)

**Table 1:** Demographics of newborns who had received high dose BCG vaccine.

Variables	n/N (%)
Age at vaccination (days),Median(Min-Max)	2.0 (2.0–4.0)
Gestational Age(weeks), Mean ± SD	38±1.2
Gender	
Male	13/26 (50)
Ward unit	
A2 well baby nursery	14/26 (53.8)
B2 well baby nursery	11/26 (42.3)
Community Health Center	1/26 (3.8)
Length of hospital stay (days), Mean ±SD	3.6±1.7
Adverse reaction developed	22/26 (84.6)
^a^ATT received	26/26 (100)
Duration of ^a^ATT(days), Median (IQR)	90 (30)
Follow-up(days),Mean ±SD	252 ± 233
No. of clinic visits, Median (IQR)	10 (7)

^a^ATT: Anti-tuberculosis treatment

**Table 2:** Adverse reactions of BCG overdose in newborns at a tertiary care hospital, Karachi.

Variables	n/N (%)
Localized skin lesions at ^a^BCG site	16/26 (61.5)
Erythema	2/16 (12.5)
Induration	3/16 (18.8)
Papule	15/16 (93.7)
Pustule	3/16 (18.7)
Jaundice	1 (3.8)
Lymphadenopathy	
Axillary	1 (3.8)
Intracranial bleeding	1 (3.8)
Outcome	
Follow-up completed for one year	11/26 (42.3)
Survived	11/11 (100)

^a^BCG: Bacillus Calmette-Guérin

**Table 3:** Clinical profile and laboratory characteristics of newborns received high dose BCG vaccine in a tertiary care hospital, Karachi.

S.No	Weight (kg)	ATT duration (Days)	Adverse Reaction	Day of life *(At which adverse event developed)*	Day of life (DOL) at which test performed/Laboratory abnormalities	Time to resolution of adverse event (days)	No. of follow up visits	Last follow-up day	Outcome
1.	2.3	97	1.5 cm papule	2	None	Not documented	8	132	No systemic disease
									Survived
2.	3.1	29	Jaundice	29	29^th^ DOL/	Lost to follow up	1	29	Lost to follow up
					Total bilirubin: 8.7 mg/dl				
					Direct bilirubin:0.9 mg/dl				
3.	3.4	134	1.0 cm papule	6	None	Not documented	12	146	No systemic disease
									Survived
4.	3.6	90	1.5 cm papule	6	None	Not documented	14	212	No systemic disease
									Survived
5.	3.8	90	1.0 cm papule	5	None	Not documented	3	46	Lost to follow-up
6.	2.7	85	1.0 cm papule	5	None	Not documented	9	149	No systemic disease
									Survived
7.	3.0	90	0.5 cm papule	7	None	Not documented	10	252	No systemic disease
									Survived
8.	3.1	90	Skin erythema	4	None	Not documented	20	676	No systemic disease
			1.0 cm pustule	8					Survived
9.	3.6	105	1.0 cm papule	6	None	Not documented	7	301	No systemic disease
									Survived
10.	2.4	81	1.0 cm papule	6	None	Not documented	7	102	No systemic disease
									Survived
11.	3.0	85	None	NA	None	NA	12	257	No systemic disease
									Survived
12.	3.0	60	1.0 cm papule	6	None	60	12	494	No systemic disease
									Survived
13.	3.0	81	1.0 cm papule	5	None	Not documented	22	327	No systemic disease
									Survived
14.	2.9	90	None	NA	None	NA	6	252	No systemic disease
									Survived
15.	3.8	90	Skin erythema and induration	12	None	1	10	249	No systemic disease
									Survived
16.	3.2	90	Post circumcision bleed with deranged coagulation	32	37^Th^DOL/	1 day after receiving vitamin K therapy	12	385	No systemic disease
					^a^PT: 42.7 seconds				Survived
					^a^INR: 4.3 ratio				
					^a^APTT:70.8 seconds				
					Factor X: 10% (Normal: 70%-152%)				
17.	3.1	90	Skin pustule	2	None	2	5	699	No systemic disease
			Intracranial Bleed	37	38^th^ DOL	355 (MRI brain performed showed resolution of hemorrhage)			
					CT Brain (Left subdural hemorrhage approximately 5.0 mm in thickness with significant mass effect and minimal subarachnoid hemorrhage in the left temporal lobe)				
					Hb: 8.3 g/dL				
					Platelet: 427* 109/L				
					^a^PT: 12.6 seconds				Survived
					^a^INR: 1.2 ratio				
					^a^APTT:29.1 seconds				
					VWF: 262% (Normal: 50%-160%)				
					Factor XIII: 102%(Normal: 70%-140%)				
18.	3.0	90	1.0 cm papule	6	None	27	17	487	No systemic disease
			Deranged coagulation	42	42^nd^ DOL	9 days after receiving vitamin K therapy *(Coagulation profile was not checked in between)*			Survived
					^a^PT: 17 seconds				
					^a^INR:1.7 ratio				
					^a^APTT:47 seconds				
19.	3.0	90	cm papule	8	None	Not documented	11	342	No systemic disease
			Unilateral axillary Lymphadenopathy (9.0 mm)	8		Not documented			Survived
20.	2.9	60	None	NA	None	NA	13	622	No systemic disease
									Survived
21.	3.3	90	1.0 cm papule	7	None	Not documented	8	232	No systemic disease
									Survived
22.	3.3	90	cm pustule	5	None	13	35	781	No systemic disease
			Persistent weeping skin lesion on left big Toe	77	90^th^ DOL/Skin punch biopsy *(inflammatory changes with eosinophilia)*	90			Survived
			Post circumcision bleed with deranged coagulation	36	36^th^ DOL	2 days after receiving vitamin k therapy			
					^a^PT 123 seconds				
					^a^INR 12.9 ratio				
					^a^APTT 140.6 seconds				
					Factor VII = 3% (67%-143%)				
					Factor IX = 10%(70%-152%)				
					Factor X = 2%(50%-163%)				
23.	3.6	90	1.0 cm induration	7	None	16	6	205	No systemic disease
									Survived
24.	3.0	90	2.0 cm papule	7	None	Not documented	14	583	No systemic disease
									Survived
25.	3.2	57	1.0 cm papule	10	None	1	14	663	No systemic disease
									Survived
26.	3.5	14	1.0 cm induration	14	None	Lost to follow-up	1	14	Lost to follow-up

^a^PT: Prothrombin Time, APTT: Activated Partial Thromboplastin Time, INR: International Normalised Ratio

## Discussion

In our study, 22/26 patients had at least one symptom following high dose BCG administration. Most of the patients (57.7%) developed a papule. 11.5% developed a pustule, 7.7% developed skin erythema, and 3.8% developed axillary lymphadenopathy. 1/26 children also developed jaundice, but he was lost-to-follow-up. None of the patients developed fever disseminated TB or osteomyelitis. These findings are in consistent with the literature in which the most common symptoms following a high dose BCG vaccine were adverse local events and few systemic complications [10]. A European report by Lotte et al. in 1984 documented most side effects of BCG overdose following immunization were exaggerated local reactions cases of large, deep ulcers, necrotic lesions or abscesses, and sometimes regional suppurative lymphadenitis were observed and only 8% developed systemic complications including fever, headache, and malaise [9, 10]. A study published in 1996 in Manchester, documented 857 cases of infants between July and November 1994 being injected intradermally with a percutaneous BCG preparation which was 5 times the upper limit of the normal dose. 61 (11%) of these infants had adverse local reactions. 48 infants (8.6%) had axillary lymphadenopathy, 6 infants had papules >10 mm, and 6 of the infants had ulcer >10 mm. One infant had an abscess which was aspirated by a needle. One infant presented at 4 months with severe combined immune deficiency (Omenn syndrome) but she did not have disseminated BCG infection [4].In the present study, it was observed that majority of the superficial local skin reactions occurred in the first 2 weeks of life which can also be seen normally with a routine BCG vaccination after birth. Almost all routine vaccine recipients experience an injection site reaction characterized by a papule which commences 2 or more weeks after immunization and then may progress to become ulcerated healing after 2–5 months leaving a superficial scar [11].However, severe local abscess and keloid formation or lymphadenitis following BCG vaccination are rarely reported and are only limited to few case reports [11].Wei et al. conducted a study in Taiwan about an event that occurred in 2012 in which the vial was diluted with 2mL instead of 3mL. That vaccine was then administered to 20 healthy infants. 3 (15%) of these infants developed injection site abscesses that resolved within 6 weeks. They all recovered without significant complications in the 18 month follow up period [12]. A case report on a 13-year-old boy who mistakenly received intramuscular (IM) intravesical BCG reported that he suffered from fever, anemia, pancytopenia, enlarged spleen, hydronephrosis and fluid around the liver. However, a relationship was not established between the symptoms and IM intravesical BCG administration. At 2 months, he developed cellulitis and swollen lymph nodes were present on sonography. The skin biopsy showed poorly formed granulomas and he was treated with isoniazid, ethambutol, and rifampicin for 6 months [13]. In a 60-year-old man 4 vials of intravesical BCG were administered intramuscularly which caused fever, headache, and sweating. He then developed local indurations and hypogastric pain after 3 days and received prophylactic isoniazid and rifampicin course [14]. The babies were followed up to a mean follow-up time of 252 days which corresponds to around 8.4 months. The median duration of ATT given was 90 days. There is no consensus on how to best manage patients with BCG overdose. Puliyel et al. in 1996 mainly managed patients by observation except for 1 patient who received abscess aspiration and 1 patient with Omenn syndrome who received ATT after diagnosis [4]. Miles and Shaw in 1996 also managed patients using observation. In the study by Ashraf, isoniazid and rifampicin were given for 2 months, another case study by Ritz et al. a surgical excision of the lump was done and then was treated with Isoniazid and Rifampicin for 6 weeks [6, 15], whereas Wei et al. in their study managed the children by only observing and following them [12].

There was one patient who suffered an intracranial bleed. This baby had a normal coagulation profile (INR of 1.2), a normal platelet count (427*10^9^/L) and also received vitamin k at birth. 3/26 (11.5%) patients had an INR of >1.0. Two of them had a post circumcision bleed. One had factor X deficiency and another kid had factor VII, IX and X deficiency which are vitamin K dependent factors. These babies responded to vitamin K therapy. This might be because of rifampicin induced coagulopathy due to vitamin K deficiency. Anti-tuberculosis medication can induce thrombocytopenia and coagulopathy which is mostly due to rifampicin [16, 17]. The pathogenesis of rifampicin-induced vitamin K deficiency is most likely to be multifactorial. It can be because of impairment of vitamin K production from gut flora due to intestinal decontamination or may be due to oxidative degradation of vitamin K as a result of hepatic microsomal enzyme stimulation [18]. Chen et al. reported rifampicin induced disseminated intravascular coagulation (DIC) in a 22 year old boy treated for pulmonary Tuberculosis at the West China Hospital [17]. Sampaziotis et al. also revealed rifampicin induced severe coagulopathy in patients with primary sclerosing cholangitis and refractory pruritus [18]. Kang et al. observed a case of a 68-year-old woman developing an acute subdural hemorrhage secondary to rifampicin induced thrombocytopenia [19]. According to the literature, rifampicin-induced thrombocytopenia usually occurs in high dose intermittent treatment because neutralizing antibodies form during continuous daily treatment [19]. It is important to start treatment early in cases of drug-induced thrombocytopenia to prevent morbidity and mortality [16]. In our study we observed coagulopathy however none of the newborns experienced thrombocytopenia. This could suggest that maybe instead of using both rifampicin and isoniazid as chemoprophylaxis; we can use only isoniazid to decrease the chance of thrombocytopenia, coagulopathy and bleeding. However, considering the nature of study and small sample size we can’t establish its temporality and causation.

There is very little data on BCG vaccination overdose in Pakistan. Moreover, medical errors tend to be underreported. By reporting these medical errors and following-up on the patients we can better understand the consequences of the medical error and their management. It also emphasizes on having checks to prevent medical errors like this in the future by increasing the awareness of health care staff. Small sample size and single centered retrospective nature are the major limitations of our study.

## Conclusion

We conclude that local adverse reactions are more common with accidental administration of high dose BCG vaccine. We recommend chemoprophylaxis for such incidents. Furthermore, vigilant follow-up, transparency and disclosure are the vital steps in the management of any medical error.
